# A preclinical animal model for evaluating the sealing capacity of covered stent grafts in acute vessel perforation

**DOI:** 10.1186/s40001-020-00429-y

**Published:** 2020-07-29

**Authors:** Alper Öner, Caroline Moerke, Anne Wolff, Sabine Kischkel, Wolfram Schmidt, Niels Grabow, Hüseyin Ince

**Affiliations:** 1 Department of Cardiology, Rostock University Medical Center, Rostock, Germany; 2 Institute for Biomedical Engineering, Rostock University Medical Center, Rostock, Germany; 3 Medizinische Klinik I im Zentrum für Innere Medizin (ZIM), Ernst-Heydemann-Str. 6, 18057 Rostock, Germany

**Keywords:** Coronary perforation, Covered stent, Stent design, Guidewire, Balloon catheter, Rupture, Oversizing, Rabbit iliac artery model

## Abstract

**Background:**

Percutaneous coronary intervention is among the most common therapeutic interventions in cardiology. This procedure may, however, be associated with a rare, though life-threatening complication: acute coronary perforation (CP). CP is primarily treated using covered stents, which are made of bare metal stents with a polytetrafluoroethylene (PTFE) or polyurethane coating. These stents’ major limitations include higher rates of thrombus formation and restenosis. Hence, there is a still unmet need for new stents regarding their design and composition. Or, to test new covered stent designs, the rabbit iliac artery has become the best-established animal model. This study sought to present a preclinical animal approach designed to test covered stents that are utilized following vessel perforation.

**Methods:**

The animal experiments were performed using New Zealand white rabbits, each weighting 3.5–4.5 kg. The animal models described herein relied on the three most common clinical causes for CP, such as guidewire-induced, balloon catheter bursting, and device oversizing. Moreover, the sealing capacity of covered stent grafts was assessed for each of these models by means of angiography.

**Results:**

We herein report a rabbit iliac artery perforation model using three different types of vessel perforation that closely mimic the clinical setting, such as guidewire-induced, balloon catheter rupture, and device oversizing. Using the same rabbit iliac perforation model, we additionally assessed the sealing capacity of a covered stent graft for each model.

**Conclusions:**

The novel rabbit iliac artery perforation models, as described in this report, represent promising animal testing approaches. While their setting is very similar to the real-life context encountered in humans, all three models are based on an animal model that is ideally suited for evaluating the sealing capacity and performance of new medical devices for humans.

## Background

One of the most common therapeutic procedures in cardiology is percutaneous coronary intervention, whose realizations have significantly increased in recent years [1]. Coronary perforation (CP) is a rare but potentially life-threatening complication, with an incidence of 0.1%–3.0% and a mortality rate exceeding 20% [2, 3]. Known CP predictors include age, female gender, complex lesions, as well as chronic total occlusion interventions or atherectomies [2, 4]. Proximal perforations are typically caused by balloons or devices, while more distal perforations are often produced by guidewires exiting the vessel lumen [5].

Although various approaches to treat CP exist, the standard method consists of using covered stents. Before covered stents were approved in the early 1990s, more than half of the patients underwent emergency cardiac surgery, which incurred a much higher mortality rate [3]. Covered stents are usually made of bare metal stents with a polytetrafluoroethylene (PTFE) or polyurethane coating. These stents display a 90% success rate for the treatment of perforation [2, 6]. Their limitations, especially in case of bulky PTFE-covered stents, include significantly higher rates of thrombus formation and restenosis, compared to regular stents [6, 7].

Hence, new developments are still needed to improve both the design and composition of covered stents. To test new covered stent designs, the rabbit iliac artery model has become one of the best-established animal models, due to its close resemblance to human coronary arteries [8, 9].

In this study, we have presented a preclinical animal approach designed to test covered stents after vessel perforation, based on the three most common clinical causes of vessel perforation: guidewire-induced, balloon catheter bursting, and device oversizing.

## Methods

### Animals and overall preparations

All animal experiments were performed according to the German animal protection guidelines that have been approved by the local animal care and use committee (7221.3-1-045/19). New Zealand white rabbits (Charles River Laboratories, Ecully, France) were employed, each of which displayed a body weight of 3.5–4.5 kg. This specific parameter ensured the carotid artery had an adequate diameter, which is essential to avoid tearing the artery upon insertion of the 4 Fr introducer sheath (Terumo, Leuven, Belgium). All animals were constantly kept at a temperature of 21 °C; humidity of 65% was maintained with a 12-h day and 12-h night rhythm, according to EU directive 2010/63/EU. Prior to the procedure, the animals were anesthetized by means of a subcutaneous injection in the neck, which consisted of a ketamine/xylazine mixture (50 mg ketamine hydrochloride [bela-pharm GmbH, Vechta, Germany] and 5 mg xylazine hydrochloride [Rompun 2%, Bayer Healthcare GmbH, Leverkusen, Germany]) per kg body weight. After 20 min, the unconscious state of the animal was deep enough to begin shaving the throat and initiate the anesthetic infusion consisting of the ketamine–xylazine mixture [50 mg ketamine and 5 mg xylazine per kg body weight added to a 100-mL sodium chloride (NaCl) infusion bag; Freeflex 100 mL NaCl 0.9, Fresenius Kabi, Bad Homburg, Germany], via the outer ear vein (26 G permanent intravenous catheter, BD Neoflon, Becton–Dickinson, Heidelberg, Germany). Throughout the experiment, the rabbit’s eyes were kept hydrated with eye gel (Vidisic, Bausch & Lomb, Berlin, Germany). Vital parameters, including pulse and oxygen saturation, were checked upon using a pulse oximeter (Nellcor, OxiMax N-65, Inspiration Medical GmbH, Bochum, Germany). Next, the animal was placed in a supine position on the operation table. Its body temperature was held at a constant 37 °C using a heating map (VetiCare, Vianen, Netherlands), and its supine position was fixed using towel rolls and leucoplast tape (BSN Medical, Hamburg, Germany).

### Surgical procedure—access to the carotid artery

The left carotid artery was exposed through a lateral skin incision of approximately 5 cm in length, which was meticulously placed 1 cm beneath the midline of the neck. After the subcutaneous tissue was dissected, the internal jugular vein became visible, lying lateral to the tracheal ring. To expose the carotid artery and vagus nerve, an incision was made in between the tracheal ring and internal jugular vein. The jugular vein, which was handled very carefully, was fixed to the surrounding tissue using a surgical hook, and it was then cautiously removed from the operating side. Bleeding was controlled by means of manual compression and cauterization. Then, the carotid artery was dissected, isolated, and verified upon by Doppler sonography (Handydrop Pro, Elcat GmbH, Wolfratshausen, Germany), before being entwined with a 3-0 Prolene suture thread (Ethlicon, Johnson & Johnson, Norderstedt, Germany) at its most distal and proximal parts. The most distal thread was used to dilate the carotid artery so as to facilitate the puncture. The proximal thread was required for fixation, anchoring, and positioning of the carotid artery, as well as for its final ligation after removing the introducer sheath. The puncture was made using a 20-G Seldinger cannula (Braun, Melsungen, Germany), which faced downward to prevent any puncture of the posterior wall of the carotid artery. After successful artery puncture, a 0.014-in. coronary guidewire (6 Fr Radiofocus Introducer Set, Terumo, Leuven, Belgium) was advanced through the lumen of the Seldinger cannula and carotid artery. After removing the Seldinger cannula, the distal and proximal threads were pulled up to prevent any hemorrhage during the change and insertion of the 4 Fr introducer sheath over the 0.014-in. coronary guidewire. The introducer sheath was pushed approximately 3–4 cm into the carotid artery and fixed using suture threads.

### Surgical procedure—perforation and stent implantation

To prevent thrombus formation, 500 international units (IU) heparin (Ratiopharm GmbH, Ulm, Germany) were injected intra-arterially, via the introducer sheath, before inserting the guidewire (ASAHI SION blue, ASAHI INTECC, The Hague, Netherlands). Under angiographic guidance (Ziehm Vario 3D, Ziehm Imaging GmbH, Nürnberg, Germany), and with the Accupaque 350 contrast medium (GE Healthcare, Braunschweig, Germany) diluted 1:1 with isotonic NaCl (Fresenius Kabi, Bad Homburg, Germany), the guidewire was advanced from the carotid artery to the proximal femoral artery and iliac artery. The guidewire’s tip was J-shaped to better cross the aortic arch and reach the descending aorta. The angle to reach the descending aorta from the left carotid artery may sometimes be very steep, and even with the J-shaped wire, we were just unable to pass it. To overcome this difficulty, the wire was guided through the aortic valve and into the left ventricle, turned into a loop, and then, sent back with the blood flow along the aortic arch into the descending aorta. The wire loop was dismantled using careful pulling movements. After angiographic evaluation of the vessel composition and iliac artery localization, the setting of the perforation was prepared. For the perforation via guidewire, a 0.014-in. Confianza PRO 12 guidewire (ASAHI INTECC, The Hague, Netherlands) was inserted, in addition to the ASAHI SION blue guidewire. The Confianza PRO 12 guidewire displays a longer and stiffer tip (20 cm vs. 3 cm for the ASHAI SION blue) and is thus better suited for performing vessel perforation. The vessel perforation was made by further movements of the Confianza PRO 12 guidewire against the distal iliac artery wall, with the vessel perforation then verified by angiography. The second option for achieving vessel perforation was carried out by overdilation and subsequent bursting of a balloon catheter (Emerge Monorail 3.0/15 mm, Boston Scientific, Marlborough, Massachusetts, USA). The balloon catheter was overdilated at 24 atm (RBP = 14 atm). The next approach designed to mimic the third clinical cause of vessel perforation was carried out using a device that was oversized for the distal iliac artery (4.5/12 mm balloon catheter, TREK, Abbott, Santa Clara, CA, USA). In addition, the oversized balloon catheter was overdilated at 24 atm until it bursted. Covered stents, which were 2.5/15 mm in size (Papyrus, Biotronik, Buelach, Switzerland), were quickly placed over the perforation site using an inflation device and an average dilation at 9 atm for 2 s (overdilation 1:1.1) (basixCOMPAK, Merit Medical, Utah, USA). Successful sealing of the perforation was checked via angiography. Then, the introducer sheath was removed, the carotid artery was ligated, and the incision was closed. The survival of the animal was monitored for 15 min after sealing the perforation. Then, the animal was gently euthanized by an intravenous injection of pentobarbital (300 mg per kg body weight, Release, WDT, Garbsen, Germany). After the animal had died, the stents were explanted and rinsed with isotonic NaCl. Then, they were fixed in 4% neutral-buffered formalin (Süsse Labortechnik GmbH, Gudensberg, Germany).

## Results

### Rabbit iliac artery perforation models

**Fig. 1:**
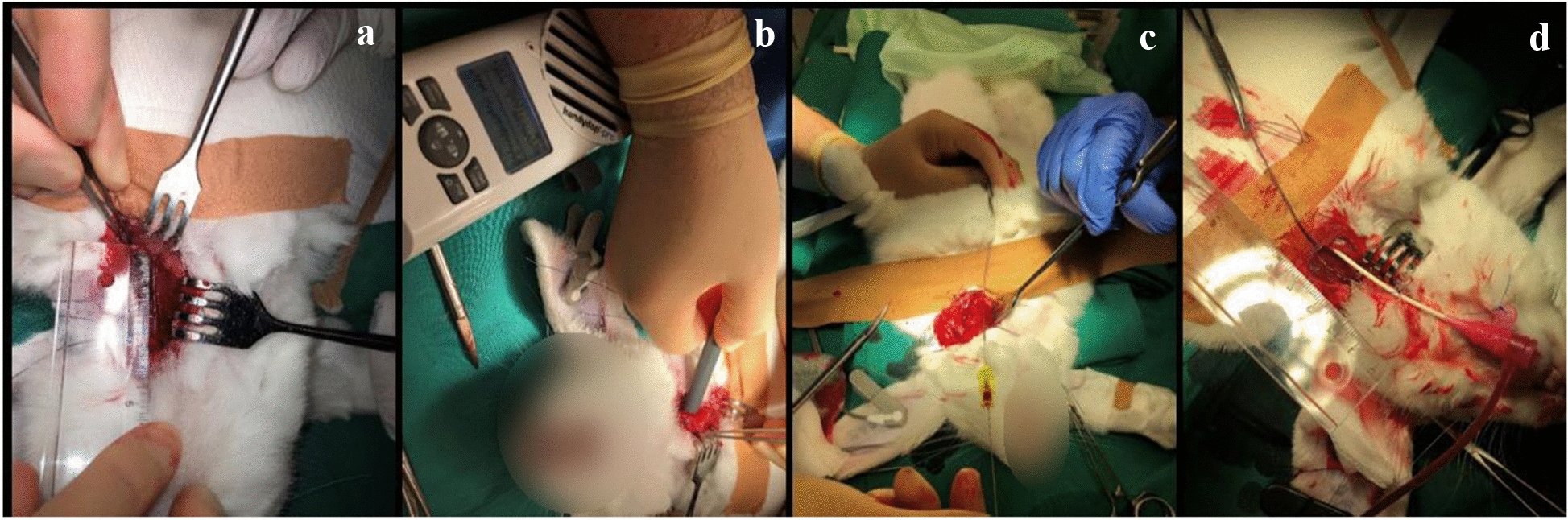
Surgical preparation of the left carotid artery of the New Zealand white rabbit for cardiac intervention. **a** Dissected, isolated, and fixed carotid artery. **b** Verification of the artery by Doppler sonography. **c** Puncture of the carotid artery by a Seldinger cannula and induction of the 0.014-in. coronary wire. **d** Fixation of the inserted 4-Fr introducer sheath

Access to the iliac artery was obtained by surgical puncture of the left carotid artery (Fig. 1). To mimic the three most common clinical causes of vessel perforation during percutaneous coronary intervention, the rabbit iliac artery was injured using a guidewire, overdilation along with the bursting of a balloon catheter, and insertion of an oversized device.

**Fig. 2:**
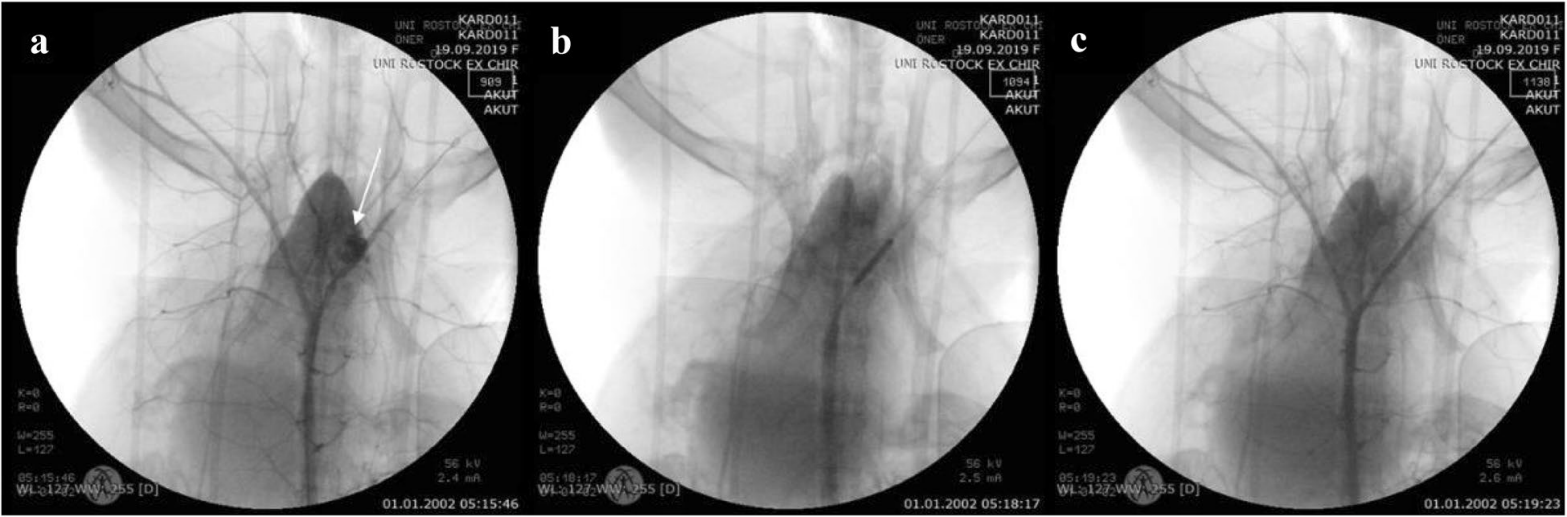
Angiography of the iliac artery perforation via guidewire. **a** Cavity filling with blood after the vessel was injured with the Confianza PRO 12 guidewire. **b** Placing of a covered stent graft over the perforation site. **c** Verification of the leakage being sealed following stent implantation

During the guidewire-induced vessel perforation, a cavity spilling of contrast media extravasation was clearly visible on angiography (Fig. 2a). After placing a covered stent graft (Fig. 2b) on the perforation site, the leakage was staunched (Fig. 2c).

**Fig. 3:**
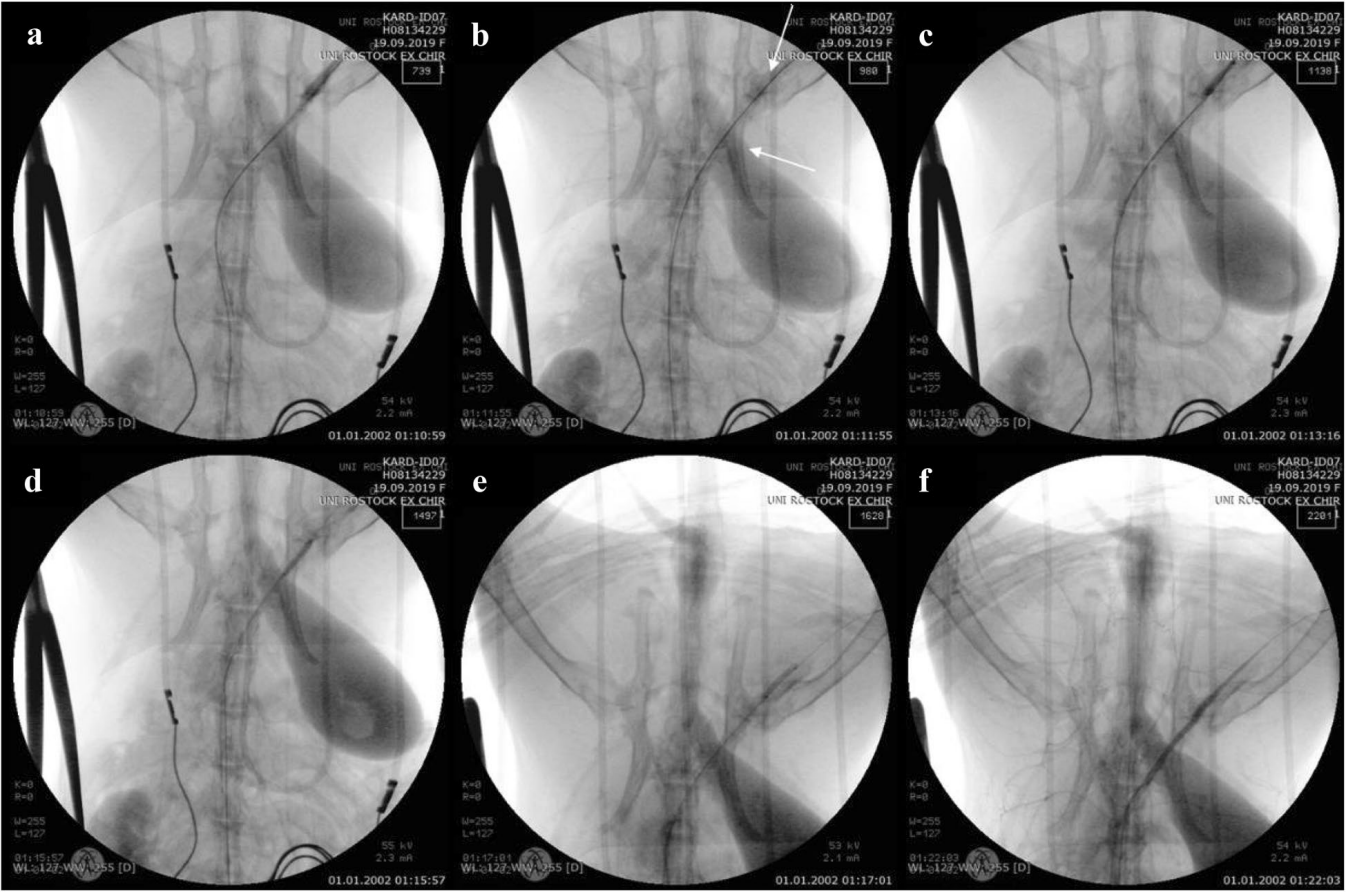
Angiography of the iliac artery perforation by overdilation and the bursting of a balloon catheter. **a** The burst of overdilated balloon catheter. **b** Leakage of blood out of the ruptured vessel. Implantation of the (**c**) first, (**d**) second, and (**e**) third covered stents. **f** Verification of the leakage being sealed after stent implantation

For the second approach to achieving vessel perforation by means of overdilation along with the bursting of a balloon catheter, the explosion of the balloon catheter (Fig. 3a) resulted in a massive artery injury and blood leakage (Fig. 3b). This rupture was fixed by implantation of three covered stent grafts (Fig. 3c–f).

**Fig. 4:**
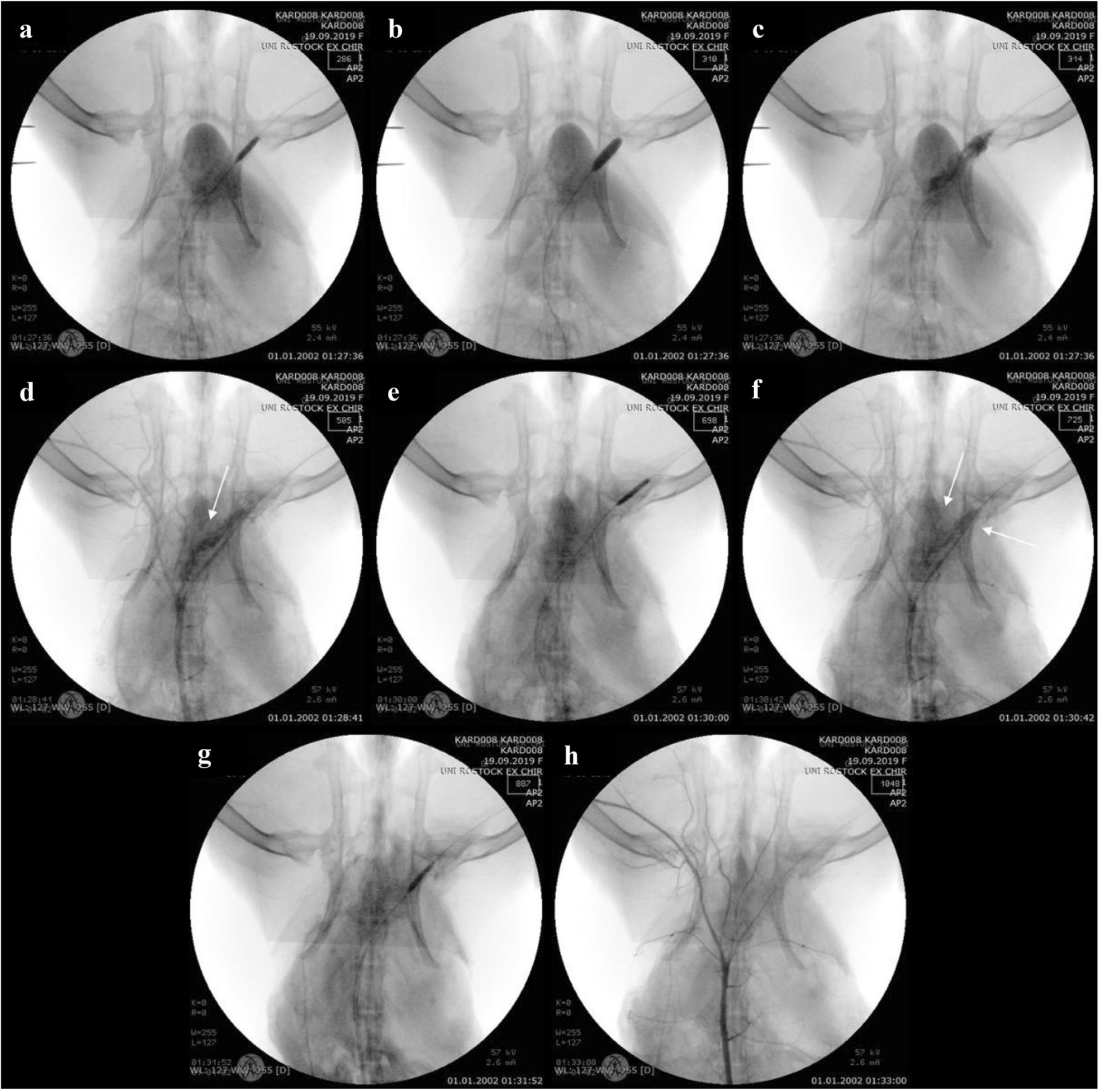
Angiography of the iliac artery perforation by overdilation and the bursting of an oversized balloon catheter. **a** Placing and dilatation of the oversized balloon catheter. **b** Dislocation of the balloon catheter after overdilation. **c** The burst oversized balloon catheter. **d** Extravasation of contrast media after vessel perforation. **e** First covered stent implantation. **f** Leakage of blood from the ruptured vessel after first stent implantation. **g** Second covered stent implantation. **h** Verification of the leakage being sealed after stent implantation

In the third method designed to attain vessel perforation by means of an oversized device, a 4.5 × 12 mm balloon catheter was inserted into the iliac artery (Fig. 4a). To intensify the perforation, the oversized balloon catheter was overdilated and slightly moved proximally (Fig. 4b) until its bursting (Fig. 4c). Massive extravasation of contrast media was the indicative of vessel perforation (Fig. 4d). After the first covered stent implantation (Fig. 4e), the extravasation of contrast media (Fig. 4f) was still visible. However, following a second covered stent implantation (Fig. 4g), the perforation was sealed, with no contrast media leakage detected upon angiography (Fig. 4h).

**Fig. 5:**
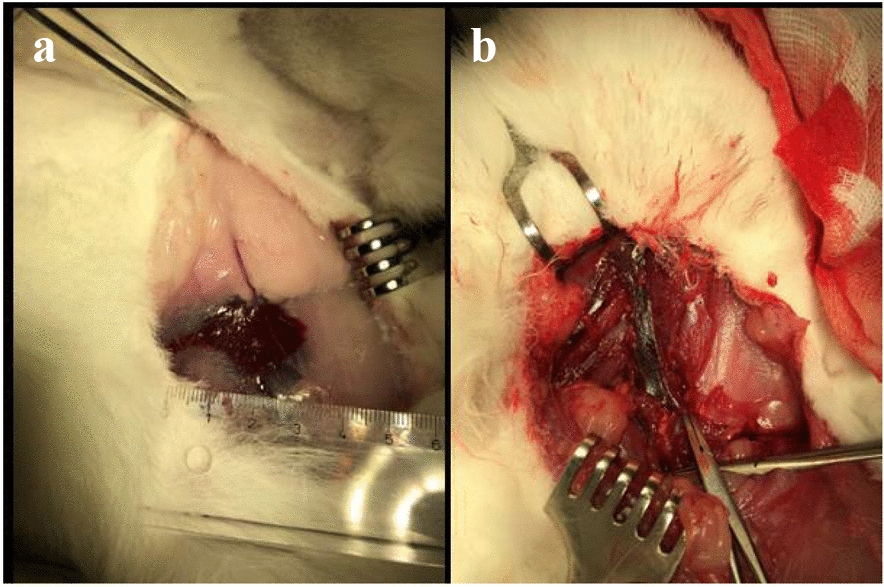
Explantation of the stents. **a** Hematoma and **b** enlarged artery after vessel perforation via overdilation and the bursting of a balloon catheter

The covered perforation was monitored for another 15 min, with the animal still alive and the perforation sealed during this time period. After the animals were euthanized, their arteries were explanted and checked again for vessel sealing by liquid flushing. For all three clinically relevant vessel perforation methods, an extended hematoma was visible within the surrounding tissues after the bleeding caused by vessel perforation (Fig. 5a). Concerning the second approach, the artery was significantly enlarged after inducing vessel perforation via balloon catheter overdilation with a 3.0/15 mm balloon catheter (Fig. 5b).

## Discussion

In this study, we have described in detail a novel approach for testing the sealing capacity of covered stent grafts in an in vivo artery perforation model. To our knowledge, this is the first study reporting on a rabbit iliac artery perforation model with vessel perforation by guidewire, balloon catheter, and oversized device, in addition to the perforation’s sealing with a covered stent graft.

The rabbit iliac model for stent implantation has been well established and is now accepted for comparing new experimental stent grafts versus conventional stents. This can essentially be explained by the similar dimensions of rabbit iliac arteries and human coronary arteries. Another standard animal model for the preclinical evaluation of endovascular devices is the porcine coronary artery model. However, this model exhibits several limitations, such as a higher granulomatous inflammatory reaction, rapid growth rate, and high body weight potential. In addition, these animals’ housing conditions are more laborious and rather expensive, compared to those of rabbits [9–11].

Recently, Taavitsainen et al. [12] published a rabbit aorta perforation model. In regard to this paper, it must, however, be mentioned that the authors punctured the aorta by means of a 2-mm biopsy punch after revealing the aorta through a midline abdominal incision. These authors succeeded in sealing the perforation with a newly developed covered stent graft. Essentially, surgically exposing the aorta after carotid artery catheterization, via surgical preparation of the carotid artery for the 6-Fr introducer sheath, proves to be very risky and dangerous for the animal. This invasive method, which is also very complicated, turns out to be a quite complex surgery with a high mortality risk.

Detailed methods have similarly been published that compare stent grafts within the scope of an atherosclerotic model in the rabbit iliac artery [11]. Conversely, our models of the iliac artery perforation using a guidewire, balloon catheter, and oversized device are all less invasive and not as dangerous. In addition, it must be stressed that our approaches fully reflect the clinical situations encountered in routine practice.

Thus, covered stent grafts must be analyzed in terms of their sealing capacity in regard to vessel perforation, as well as in terms of their performance concerning endothelization rates, inflammatory reactions, thrombosis, and restenosis formation after implantation. Long-term patency rates are deemed poor, particularly for conventional PTFE-covered stents [6, 7]. This further emphasizes the imperative need for new developments and appropriate testing models.

To end, we would like to further emphasize the prerequisite of having appropriate animal models for biomedical research. To be appropriate, an animal model must first of all be relevant, meaning that a phenomenon to be studied in the animal must be as close as possible to the phenomenon that is encountered in humans. The models presented herein appear to fulfill these requirements. Accordingly, we assume that these novel approaches may play a catalytic role in our search for superior stent grafts.

## Conclusions

Based on these herein reported rabbit iliac artery perforation models, we present testing scenarios that nearly replicate the clinical situations encountered in routine practice, using an animal model that is suitable for evaluating the sealing capacity and performance of new medical devices for humans.

## Data Availability

The datasets used and/or analyzed during the current study are available from the corresponding author on reasonable request.

## Abbreviations

CP: Coronary perforation
IU: International units
NaCl: Sodium chloride
PTFE: Polytetrafluoroethylene
